# Masticatory movements and food textures in older patients with eating difficulties

**DOI:** 10.1111/ger.12596

**Published:** 2021-10-21

**Authors:** Yoko Kato, Takeshi Kikutani, Takashi Tohara, Noriaki Takahashi, Fumiyo Tamura

**Affiliations:** ^1^ Division of Clinical Oral Rehabilitation Graduate School of Life Dentistry The Nippon Dental University at Tokyo Japan; ^2^ Division of Rehabilitation for Speech and Swallowing Disorders Tama Oral Rehabilitation Clinic The Nippon Dental University at Tokyo Japan

**Keywords:** appropriate food, elderly people, jaw movement, masticatory function, oral motor function

## Abstract

**Objectives:**

To investigate whether masticatory movements in older patients with eating difficulties were associated with oral motor function, physical function, and appropriate food textures.

**Background:**

There are few reports on the association between masticatory movements and food textures in older patients with eating difficulties.

**Materials and Methods:**

This cross‐sectional study involved outpatients at a clinic that specialised in eating and swallowing rehabilitation. Masticatory movements were evaluated as normal or abnormal masticatory path patterns. Oral and physical functions were assessed in terms of oral and physical status, muscle strength and motor skills. The appropriate food texture was determined based on fibreoptic endoscopic evaluation of swallowing and a video fluoroscopic swallowing study. The associations between food texture and masticatory organ, muscle strength and motor skills were analysed.

**Results:**

A total of 126 outpatients (75 men and 51 women; mean age, 78.2 years; SD, 9.6 years) were included in the analysis. 68 participants (54.0%) showed abnormal masticatory movements. Masticatory movement was associated with masticatory performance (odds ratio [OR] = 0.99, 95% confidence interval [CI] = 0.98‐0.99), oral diadochokinesis (OR = 0.55, CI = 0.35‐0.86) and stepping test (OR = 0.92, CI = 0.86‐0.97). Masticatory movement (OR = 2.94, CI = 1.23‐7.01) and the number of natural teeth (OR = 0.94, CI = 0.89‐0.99) were associated with normal food.

**Conclusion:**

Masticatory movements in older patients with eating difficulties may be associated with appropriate food textures whilst being influenced by individual differences in systemic motor control. Masticatory movements may be as important as teeth to enjoy eating.

## 1 INTRODUCTION

For older adults, food texture is an important issue that may influence their quality of life,^1^ nutritional status^2^, ^3^, ^4^, ^5^ and prognosis.^6^, ^7^ The greater the variety of foods they can chew, the greater the satisfaction with their diet. This has a positive impact on oral health‐related quality of life and subjective well‐being.^1^ Normal types of foods may contain hard textures and fibres, which may be improperly or inadequately chewed, thereby increasing the choking risk of people with poor masticatory function.^6^ Moreover, avoiding certain foods that are difficult to chew and limiting the variety of foods consumed can lead to nutritional deficiencies.^2^, ^3^, ^4^ Texture‐modified diets allow the continuation of safe oral intake whilst minimising the choking risks,^8^ but they generally contain lower energy and protein than normal foods,^9^, ^10^ which may increase the risk of malnutrition,^5^ skeletal muscle loss,^10^ and a worse overall prognosis.^7^ To enjoy eating with as little risk as possible, it is necessary to evaluate a person's masticatory function from various aspects and carefully select an appropriate food texture.

Previous reports^2^, ^3^ on masticatory function and ingestible food texture have mainly focused on teeth and occlusion as the organic factors of mastication, such as the association between the loss of teeth and occlusion and the limited variety of food intake. One of the few studies^11^ on the motor factors of mastication and ingestible food texture reported that tongue pressure was higher in regular food consumers than in those on a texture‐modified diet. Mastication is a process involving multiple factors, including teeth and occlusion as masticatory organs, jaw and tongue movement as oral motor skills, oral sensations,^12^ and neural mechanisms that control movement.^13^ A recent study^14^ reported that tongue pressure and oral diadochokinesis were associated with masticatory performance in healthy older adults with 28 teeth. This suggests that not only teeth but also oral motor skills are important for masticatory function.

Jaw movement during mastication, also referred to as a masticatory movement, is coordinated with the tongue, hyoid bone and soft palate.^15^ Masticatory movement is associated with masticatory performance,^16^ and efficient jaw movement allows for the proper processing of food. Abnormal masticatory movements have been considered due to peripheral feedback from the oral cavity, such as the periodontal ligament,^17^ occlusion^18^, ^19^ and dental prosthesis.^20^ A recent neuroimaging study reported that the brain works differently during mastication in older adults than in younger adults.^21^ Another study^22^ suggested that the brain influences individual differences in masticatory performance in healthy older adults. The structural features of the brain indicated by grey matter volume, and the functional features of the brain by the resting‐state functional connectivity were associated with masticatory performance. Based on the above, it is possible that in older adults, brain characteristics and individual differences may modify masticatory movements through the motor control system. In such cases, the influence on motor skills may appear in the body region as well as in the oral region. It is also possible that different masticatory movements may require different textures to be eaten.

This study aimed to investigate whether masticatory movements in older patients with eating difficulties are associated with oral motor function, physical function and suitable food textures.

## 2 MATERIALS AND METHODS

The study was approved by the Ethics Committee of the Nippon Dental University, School of Life and Dentistry (approval number NDU‐T2018‐35). Before participating, informed consent was obtained from each participant. If the participant had writing difficulty, a family member or caregiver signed the patient's name on their behalf, along with their signature.

### 2.1 Study design and setting

This cross‐sectional study was conducted in a clinic specialising in eating and swallowing rehabilitation on the same day each week between April 2019 and March 2021.

### 2.2 Participants

The baseline participants were 152 outpatients, aged 50 years or over. Some patients received enteral feeding through nasogastric tubes or percutaneous endoscopic gastrostomy. Since patients on enteral feeding included those with severe dysphagia, the selection criteria were based on safety considerations, that is, only patients on oral intake were included, and not those on enteral feeding. Patients with oral cancer were excluded because of possible masticatory disorders due to loss of normal morphology of the tongue, palate, and jaw crest. Patients without occlusal support by natural or artificial teeth were excluded because of possible masticatory disorders caused by the absence of chewing teeth. Patients with severe dementia, choking episodes, tooth or jaw pain or paralysis of the lower limbs were also excluded because of the difficulty in performing the examination.

### 2.3 Evaluations

A medical questionnaire was used to collect basic information such as gender, age and underlying medical conditions. Height and weight were measured, and the body mass index (BMI) was calculated by dividing weight (kg) by the square of height (m).

#### 2.3.1 The number of teeth

The number of natural teeth was defined as the number of natural or treated teeth. Severe mobility teeth as Miller's index class 3 grade^23^ and residual roots were excluded considering their small contribution to occlusion. The number of functional teeth was defined as the number of natural teeth and artificial teeth such as pontic, dentures and implant‐supported prostheses.

#### 2.3.2 Masticatory movement

**FIGURE 1:**
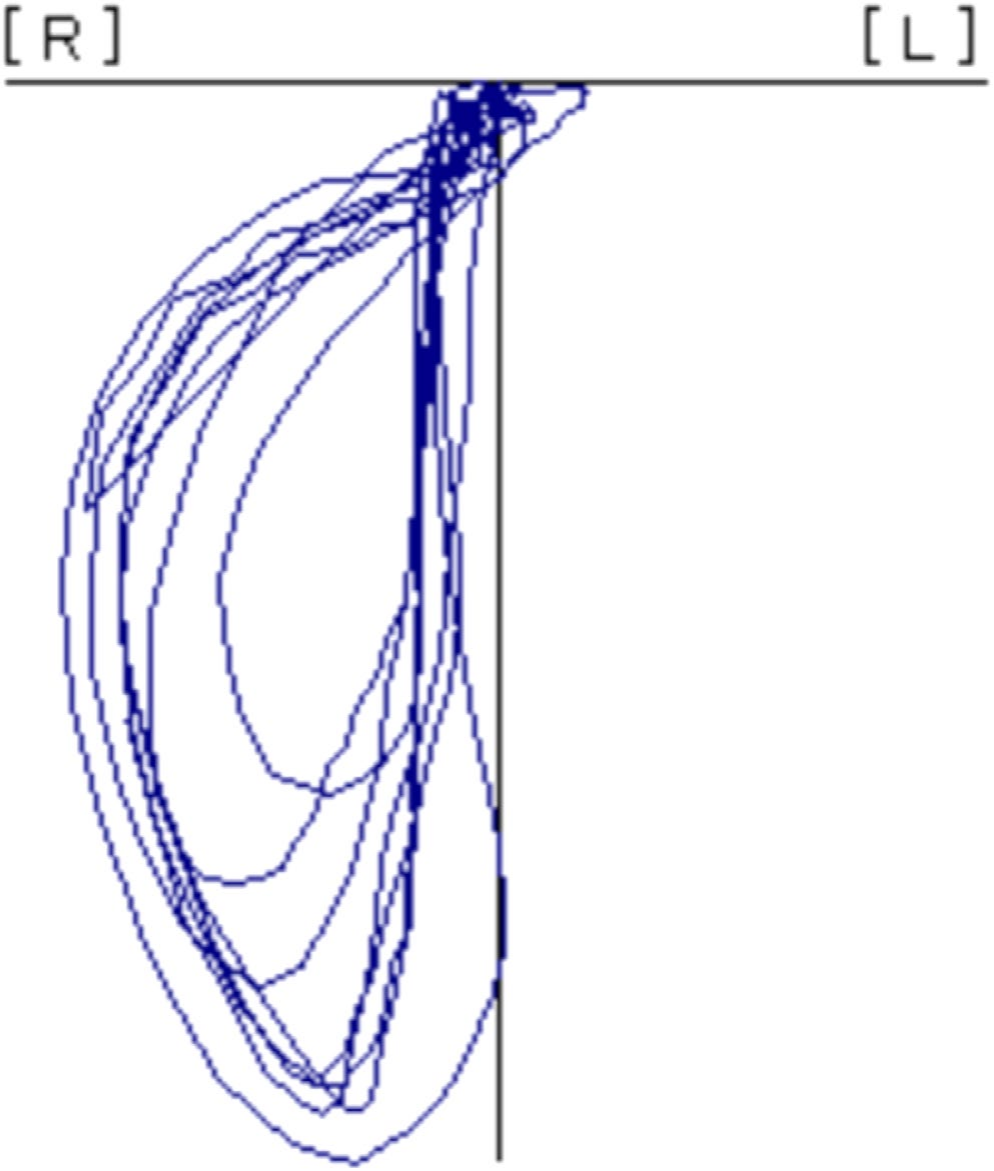
The masticatory path pattern at the 5th to 14th cycle

The masticatory path pattern was used as an index of masticatory movement and as an index of masticatory motor skill. The measurement and classification followed the method described by Kobayashi et al.^18^ A jaw movement recording device (Motion VISI‐TRAINER V‐1; GC Corporation) and gummy jelly (GLUCOLUMN; GC Corporation) were used. Participants chewed the gummy jelly for 20 s on their habitual chewing side. The trajectory of jaw movement was recorded three‐dimensionally, and the 5th to 14th cycles were ranged using dedicated software. The superimposed pattern (Figure 1) was displayed and classified into seven patterns by Kobayashi et al,^18^ which are defined by the characteristics of the path. Seven patterns include six combinations of three types of opening paths (a linear or concave path, a path toward the chewing side after toward the non‐working side and a convex path) and two types of closing paths (a convex path and a concave path), plus one crossed pattern of opening and closing paths. Of these patterns, patterns 1 and 3 were considered healthy or normal patterns, whilst five patterns other than 1 and 3 were considered abnormal patterns.^18^, ^20^ In this study, participants were divided into two groups according to normal or abnormal mastication patterns.

#### 2.3.3 Masticatory performance

Glucose‐containing gummy jelly (GLUCOLUMN; GC Corporation), and a glucose‐measuring device (GLUCO SENSOR GS‐II; GC Corporation) were used. According to the method of Shiga et al,^24^ participants chewed the gummy jelly for 20 s on the habitual chewing side, rinsed their mouths lightly with 10 ml of distilled water, and spat out all chewed gummy, saliva and water into a filtered cup. The glucose concentration (mg/dl) in the filtrate was used as the measurement value.

#### 2.3.4 Oral diadochokinesis (ODK)

Oral diadochokinesis (ODK) is a comprehensive measurement of tongue‐lip motor speed and dexterity. In this study, ODK was used as a parameter of tongue and lip motor skills. The participants repeated the monosyllables /pa/, /ta/, or /ka/ for 5 s as quickly as possible. The number of syllables produced per s was calculated using an automatic counter (Kenko‐Kun Handy; Takei Scientific Instruments Co., Ltd.).^25^ In this study, the mean scores of /pa/, /ta/, and /ka/ were used as representative values for the tongue and lip motor skills for the statistical analysis.

#### 2.3.5 Tongue pressure

Tongue pressure was used as an index of tongue muscle strength.^26^ A tongue pressure measurement device (JM‐TPM; JMS Co., Ltd.) was used. Following the method of Hayashi et al,^27^ the participant pressed the balloon probe against the hard palate with a maximum force of 2 s. The test was repeated three times at 1 min intervals, and the peak value was recorded as the score.

#### 2.3.6 Stepping test

The stepping test is one of the physical fitness measurements that Kimura et al^28^ have revised considering the safety of older adults who have not exercised for a long time. It assesses physical agility by opening and closing movements of the lower limbs in a seated position. The participant sat in a chair with their legs easily movable and grasped the seat with both hands to stabilise the upper body. Two lines were drawn 30‐cm apart on the floor at their feet, and the starting position was with both feet inside the lines. The participant opened and closed their legs for 20 s without stepping on the line as quickly as possible. Their feet alternately touched the outside and inside of the lines. The number of times both feet touched the inside of the two lines without stepping on the lines or dragging their feet was counted and recorded.

#### 2.3.7 Grip strength

A digital dynamometer (TKK‐5401; Takei Scientific Instruments Co., Ltd.) was used. The grip width was adjusted so that the second joint of the index finger was almost at a right angle. Participants who could stand alone without support were measured in a standing position, whilst those who had difficulty standing were seated for safety. Participants held the grip with their arms extended below and with the maximum force. The measurement was performed once on each side, and the higher value was recorded.

#### 2.3.8 Food texture

Appropriate food texture for each patient was determined by fibreoptic endoscopic evaluation of swallowing and video fluoroscopic swallowing study. Patients were asked to bite the test food of different textures to check for bolus formation, laryngeal penetration or aspiration, and severity of pharyngeal residue. The texture that minimised the risk of aspiration or choking was determined to be appropriate for the patient's function. The food texture groups were divided into three categories according to a previous study by Shimizu et al^10^ into a normal food (Regular), almost normal texture, but with specific food limitations (Limited), and texture‐modified diets based on the Japanese Dysphagia Diet Criteria classification (Modified).

### 2.4 Sample size calculation

The sample size was calculated using G*Power 3.1.9.2 Statistical Power Analyses (Heinrich‐Heine‐Universität Düsseldorf) with an alpha error of 0.05, a power of 0.8, and a medium effect size of 0.5.^29^ More than 106 participants were estimated to be required in this study.

### 2.5 Statistical analysis

The student's *t*‐test and the Mann‐Whitney *U*‐test were used to compare the oral and physical evaluations between the masticatory movement groups. Logistic regression analysis was performed to investigate the oral and physical items associated with masticatory movement. The chi‐square test was used to compare the oral evaluations among the food texture groups. Logistic regression analysis was performed using the number of natural teeth, masticatory movements and tongue pressure as explanatory variables to investigate whether masticatory items associated with differences in texture were masticatory organs, motor skills or muscle strength. The objective variables were food variety and texture limitations. Missing values were replaced by mean values. The level of significance was set at *P* < .05. All statistical analyses were performed using SPSS, version 26.0 (IBM SPSS Statistics for Windows, Version 26.0; IBM Corp.).

## 3 RESULTS

**FIGURE 2:**
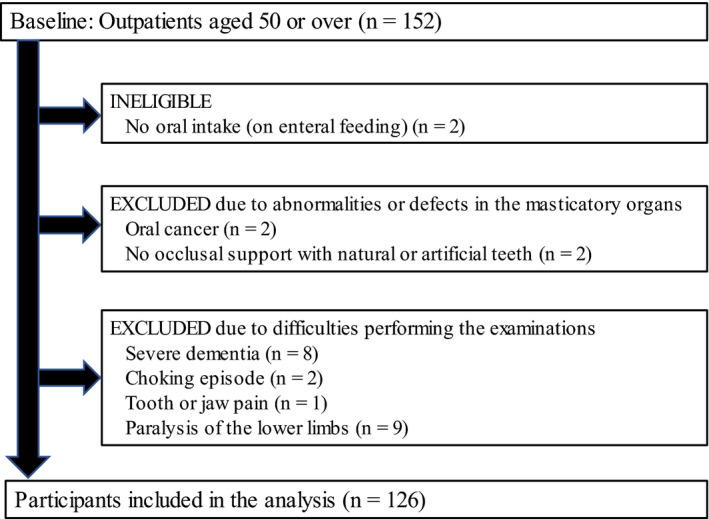
Flow chart of the participants

A total of 126 participants (75 men, 51 women; mean age, 78.2 years; SD 9.6 years) were included in the analysis. A flowchart of the study is shown in Figure 2. The general characteristics of the participants were as follows: male 75 (59.6%), aged 79.5 years (interquartile range [IQR], 72.75‐85.00 years), the number of natural teeth 21.5 (IQR, 12.00‐26.00), the number of functional teeth 28.0 (IQR, 26.00‐28.99), the BMI 21.5; SD 3.6 kg/m^2^. The primary diseases of the participants were as follows: neuromuscular disease, 23 (18.3%); dementia, 4 (3.2%); cerebrovascular disease, 41 (32.5%); gastrointestinal disease, 13 (10.3%); vagus nerve disorder, 3 (2.4%); psychiatric disease, 16 (12.7%); sarcopenia, 6 (4.8%); and others, 20 (15.9%). Additionally, there were two missing grip strength values.

Table 1 shows a comparison of the oral and physical evaluations between the masticatory movement groups. The abnormal group showed lower values in BMI, masticatory performance, ODK, tongue pressure, stepping test and grip strength than the normal group. The presence or absence of each disease was not associated with masticatory movements.

**TABLE 1:** Comparison of oral and physical evaluations between masticatory movement groups

	Masticatory movement		*P‐*value
	Normal (n = 58)	Abnormal (n = 68)	
Gender, men n (%)	32 (55.2)	43 (63.2)	.358^c^
Age (y), median [IQR]	78.0 [70.00‐84.00]	80.5 [75.00‐86.75]	.163^a^
BMI (kg/m^2^), mean (SD)	22.4 (3.3)	20.8 (3.7)	.017^b^
Primary disease			
Neuromuscular disease, n (%)	10 (17.2)	13 (19.1)	.786^c^
Dementia, n (%)	1 (1.7)	3 (4.4)	.391^c^
Cerebrovascular diseases, n (%)	16 (27.6)	25 (36.8)	.273^c^
Gastrointestinal diseases, n (%)	6 (10.3)	7 (10.3)	.993^c^
Vagus nerve disorders, n (%)	1 (1.7)	2 (2.9)	.655^c^
Psychiatric diseases, n (%)	11 (19.0)	5 (7.4)	.051^c^
Sarcopenia, n (%)	3 (5.2)	3 (4.4)	.842^c^
Other, n (%)	10(17.2)	10(14.7)	.698^c^
Number of natural teeth, median [IQR]	24.0 [16.00‐27.00]	20.0 [8.25‐25.00]	.066^a^
Number of functional teeth, median [IQR]	27.0 [26.00‐28.00]	28.0 [25.00‐28.00]	.938^a^
Masticatory performance (mg/dL), median [IQR]	172.0 [142.75‐225.50]	121.0 [80.50‐157.00]	<.001^a^
ODK (times/s), median [IQR]	5.9 [5.34‐6.40]	4.8 [3.73‐5.85]	<.001^a^
Tongue pressure (kPa), mean (SD)	31.8 (9.1)	23.7 (9.1)	<.001^b^
Stepping test (times/20 s), median [IQR]	26.0 [22.75‐31.00]	21.0 [18.00‐24.75]	<.001^a^
Grip strength (kg), mean (SD)	27.7 (8.8)	23.8 (8.6)	.014^b^

Abbreviations: BMI, body mass index; IQR, interquartile range; ODK: oral diadochokinesis; SD: standard deviation.

^a^ Mann‐Whitney *U‐*test.

^b^ *t*‐test.

^c^ Chi‐square test.

The logistic regression analysis of oral evaluations associated with masticatory movement is shown in Table 2. The masticatory movements were associated with masticatory performance (odds ratio [OR] = 0.99, 95% confidence interval [CI] = 0.98‐0.99) and ODK (OR = 0.55, 95% CI = 0.35‐0.86). The number of natural teeth and tongue pressure was not associated with masticatory movement.

**TABLE 2:** Logistic regression analysis of oral evaluations associated with masticatory movement

Independent variables	*B*	OR	95% CI	*P‐*value
Gender (men = 1, women = 2)	−0.490	0.61	0.26‐1.45	.266
Age (y)	−0.009	0.99	0.95‐1.04	.709
Number of natural teeth	−0.008	1.01	0.96‐1.06	.761
Masticatory performance (mg/dL)	−0.011	0.99	0.98‐0.99	.012
ODK (times/s)	−0.605	0.55	0.35‐0.86	.008
Tongue pressure (kPa)	−0.054	0.95	0.89‐1.00	.065

Abbreviations: CI, confidence interval; ODK, oral diadochokinesis; OR, odds ratio.

The logistic regression analysis of the physical evaluations associated with masticatory movement is shown in Table 3. The masticatory movement was associated with the stepping test (OR = 0.92, 95% CI = 0.86‐0.97), but not with BMI and grip strength.

**TABLE 3:** Logistic regression analysis of physical evaluations associated with masticatory movement

Independent variables	*B*	OR	95% CI	*P‐*value
Gender (men = 1, women = 2)	−0.706	0.49	0.19‐1.30	.152
Age (y)	−0.002	1.00	0.96‐1.04	.929
BMI (kg/m^2^)	−0.108	0.90	0.80‐1.01	.072
Stepping test (times/20 s)	−0.088	0.92	0.86‐0.97	.004
Grip strength (kg)	−0.046	0.96	0.90‐1.01	.135

Abbreviations: BMI, body mass index; CI, confidence interval; OR, odds ratio.

Table 4 shows a comparison of oral evaluations among the food texture groups. The proportion of the normal masticatory movement group decreased with a more limited food texture. Other oral evaluations seemed to decrease in the median and mean values, with the limitation of the food texture, but they were not associated with masticatory movement in the chi‐square test.

**TABLE 4:** Comparison of the oral evaluations among food texture groups

	Food texture groups			*P‐*value^a^
	Regular (n = 44)	Limited (n = 56)	Modified (n = 26)	
Gender, men n (%)	26 (59.1)	32 (57.1)	17 (65.4)	.777
Age (y), median [IQR]	78.5 [69.50‐83.00]	79.0 [71.25‐86.75]	81.5 [77.75‐85.25]	.506
Number of natural teeth, median [IQR]	25.0 [17.25‐27.00]	21.0 [9.00‐26.00]	16.5 [7.50‐21.75]	.460
Tongue pressure (kPa), mean (SD)	31.3 (10.5)	27.6 (7.9)	20.6 (9.6)	.421
Masticatory performance (mg/dL), median [IQR]	167.0 [135.25‐218.50]	142.5 [108.50‐206.00]	95.5 [68.00‐153.50]	.592
ODK (times/s), median [IQR]	6.1 [5.23‐6.45]	5.3 [4.33‐5.83]	4.7 [3.68‐5.69]	.268
Masticatory movement, normal n (%)	30 (68.2)	19 (33.9)	9 (34.6)	.001

Abbreviations: IQR, interquartile range; ODK, oral diadochokinesis; SD, standard deviation.

^a^ Chi‐square test.

The logistic regression analysis of mastication items associated with Regular is shown in Table 5. The number of natural teeth (OR = 1.06, 95% CI = 1.01‐1.12) and masticatory movement (OR = 2.94, 95% CI = 1.23‐7.01) were associated with Regular.

**TABLE 5:** Logistic regression analysis of mastication items associated with regular

Independent variables	*B*	OR	95% CI	*P‐*value
Gender (men = 1, women = 0)	0.033	1.03	0.45‐2.38	.939
Age (y)	−0.004	1.00	0.95‐1.04	.849
Number of natural teeth	0.061	1.06	1.01‐1.12	.026
Masticatory movement (normal = 1, abnormal = 0)	1.078	2.94	1.23‐7.01	.015
Tongue pressure (kPa)	0.036	1.04	0.99‐1.09	.148

Abbreviations: CI, confidence interval; OR, odds ratio.

The logistic regression analysis of mastication items associated with Modified is shown in Table 6. Tongue pressure (OR = 0.90, 95% CI = 0.84‐0.96) was associated with modified, whereas the number of natural teeth and masticatory movement were not associated.

**TABLE 6:** Logistic regression analysis of mastication items associated with modified

Independent variables	*B*	OR	95% CI	*P‐*value
Gender (men = 1, women = 0)	0.436	1.55	0.57‐4.19	.391
Age (y)	0.012	1.01	0.96‐1.07	.678
Number of natural teeth	−0.035	0.97	0.92‐1.02	.191
Masticatory movement (normal = 1, abnormal = 0)	0.389	1.48	0.49‐4.41	.486
Tongue pressure (kPa)	‐0.110	0.90	0.84‐0.96	.001

Abbreviations: CI, confidence interval; OR, odds ratio.

## 4 DISCUSSION

Masticatory movements were associated with the ability to consume regular food whilst being associated with oral and physical motor skills. The results suggest that masticatory movements may be an important factor in determining the appropriate food texture in patients with eating difficulties.

The percentage of abnormal masticatory path patterns has been reported as 25% in healthy young adults.^18^ In the present study, the result was 54%, which is approximately twice as high. Previous studies^17^, ^18^, ^19^, ^20^ reported that oral conditions, such as teeth, occlusion and dentures, are associated with abnormal masticatory movements. In this study, oral status, such as the number of natural teeth and functional teeth were not associated with masticatory movements. The masticatory movement was associated with oral and physical motor skills. The association of masticatory movement with physical motor function and oral motor function suggests that abnormal masticatory movement may be influenced by systemic motor control.

The reasons for systemic influences may include the older age of the participants and the underlying medical conditions. As the participants were biased towards symptomatic patients rather than healthy older adults, the systemic influence of simply being older is unknown. Some participants had diseases that may influence motor function, such as cerebrovascular disease, neuromuscular disease and dementia. However, this study did not find any association between disease and masticatory movement. The result could be because the disease severity of the subjects was mild enough to allow oral intake, or because the small number of cases per disease made it difficult to detect differences.

In the analysis of masticatory function and appropriate food texture, the masticatory movement was associated with normal food, as was the number of natural teeth. Both teeth and motor skills seem to be necessary to enjoy eating without the limitation of food textures. Texture‐modified diets were associated with tongue pressure, supporting previous reports,^11^ but not with masticatory movements.

This study investigated the association between masticatory movements and appropriate food textures in older adults, which has not been widely reported. Together with its association with oral and physical motor functions, this study could be one of the few studies that investigated masticatory movements in older adults from various aspects.

There were some limitations to this study. First, as this was a cross‐sectional study, the causal association between each factor is unknown. It is necessary to examine causal associations through future longitudinal studies. Second, there was bias in the sample. The participants were patients who had visited a specialist clinic complaining of eating difficulties, so they were a group who already had some subjective symptoms of masticatory concerns. Therefore, the proportion of people with poor masticatory function could be higher than that in the general older population. In the future, intervention into the oral movements of older adults should be carried out to determine whether oral movements improve the masticatory movements and food textures that can be consumed.

## 5 CONCLUSION

Masticatory movements in older adults with eating difficulties may be associated with appropriate food textures whilst being influenced by individual differences in systemic motor control. Masticatory movement may be as important as teeth for eating enjoyment without limiting the variety of food.

## CONFLICT OF INTEREST

The authors declare no conflict of interest.

## AUTHOR CONTRIBUTIONS

Yoko Kato and Takeshi Kikutani conceptualised the study, curated the data for analysis, administered the project, and visualised the data. Yoko Kato, Takeshi Kikutani and Takashi Tohara were involved in the data analysis and provided the resources. Yoko Kato and Takeshi Kikutani investigated the study and were involved in statistical analysis. Noriaki Takahashi supervised the study. Fumiyo Tamura reviewed and edited the manuscript. Yoko Kato wrote the original draft of the manuscript.

## Data Availability

The data are not publicly available as patient consent was obtained under the condition to use only for this study.

## References

[1] Iinuma T , Arai Y , Takayama M , et al. Satisfaction with dietary life affects oral health‐related quality of life and subjective well‐being in very elderly people. J Oral Sci. 2017;59(2):207‐213. 10.2334/josnusd.16-0414 28637980

[2] Sheiham A , Steele JG , Marcenes W , et al. The relationship among dental status, nutrient intake, and nutritional status in older people. J Dent Res. 2001;80(2):408‐413. 11332523 10.1177/00220345010800020201

[3] Yoshida M , Kikutani T , Yoshikawa M , Tsuga K , Kimura M , Akagawa Y . Correlation between dental and nutritional status in community‐dwelling elderly Japanese. Geriatr Gerontol Int. 2011;11(3):315‐319. 10.1111/j.1447-0594.2010.00688.x 21265972

[4] Motokawa K , Mikami Y , Shirobe M , et al. Relationship between chewing ability and nutritional status in Japanese older adults: a cross‐sectional study. Int J Environ Res Public Health. 2021;18(3):1216. 10.3390/ijerph18031216 33572969 PMC7908427

[5] Jensen GL , Mirtallo J , Compher C , et al. Adult starvation and disease‐related malnutrition: a proposal for etiology‐based diagnosis in the clinical practice setting from the international consensus guideline committee. J Parenter Enteral Nutr. 2010;34(2):156‐159. 10.1177/0148607110361910 20375423

[6] Cichero JAY , Steele C , Duivestein J , et al. The need for international terminology and definitions for texture‐modified foods and thickened liquids used in dysphagia management: foundations of a global initiative. Curr Phys Med Rehabil Rep. 2013;1:280‐291. 24392282 10.1007/s40141-013-0024-z PMC3873065

[7] Maeda K , Ishida Y , Nonogaki T , et al. Burden of premorbid consumption of texture modified diets in daily life on nutritional status and outcomes of hospitalization. J Nutr Health Aging. 2019;23(10):973‐978.3178172710.1007/s12603-019-1237-3

[8] Pardoe EM . Development of a multistage diet for dysphagia. J Am Diet Assoc. 1993;93(5):568‐571. 8315168 10.1016/0002-8223(93)91819-c

[9] Nowson CA , Sherwin AJ , McPhee JG , Wark JD , Flicker L . Energy, protein, calcium, vitamin D and fibre intakes from meals in residential care establishments in Australia. Asia Pac J Clin Nutr. 2003;12(2):172‐177. 12810407

[10] Shimizu A , Maeda K , Tanaka K , Ogawa M , Kayashita J . Texture‐modified diets are associated with decreased muscle mass in older adults admitted to a rehabilitation ward. Geriatr Gerontol Int. 2018;18(5):698‐704. 29278280 10.1111/ggi.13233

[11] Tanaka Y , Nakano Y , Yokoo M , Takeda Y , Yamada K , Kayashita J . Examination about the relation of meal form, tongue pressure, grip and walking state in inpatient and elderly residents. Jpn J Dysphagia Rehabil. 2015;19(1):52‐62.

[12] Ikebe K , Amemiya M , Morii K , et al. Association between oral stereognostic ability and masticatory performance in aged complete denture wearers. Int J Prosthodont. 2007;20(3):245‐250. 17580454

[13] Morquette P , Lavoie R , Fhima MD , Lamoureux X , Verdier D , Kolta A . Generation of the masticatory central pattern and its modulation by sensory feedback. Prog Neurogibol. 2012;96(3):340‐355. 10.1016/j.pneurobio.2012.01.011 22342735

[14] Sagawa K , Furuya H , Ohara Y , et al. Tongue function is important for masticatory performance in the healthy elderly: a cross‐sectional survey of community‐dwelling elderly. J Prosthodont Res. 2019;63(1):31‐34. 10.1016/j.jpor.2018.03.006 30197226

[15] Mioche L , Hiiemae KM , Palmer JB . A postero‐anterior videofluorographic study of the intra‐oral management of food in man. Arch Oral Biol. 2002;47(4):267‐280. 11922870 10.1016/s0003-9969(02)00007-9

[16] Uesugi H , Shiga H . Relationship between masticatory performance using a gummy jelly and masticatory movement. J Prosthodont Res. 2017;61(4):419‐425. 28237698 10.1016/j.jpor.2017.01.001

[17] Trulsson M , Johansson RS . Encoding of tooth loads by human periodontal afferents and their role in jaw motor control. Prog Neurogibol. 1996;49(3):267‐284. 10.1016/s0301-0082(96)00016-0 8878305

[18] Kobayashi Y , Shiga H , Arakawa I , Yokoyama M , Nakajima K . Masticatory path pattern during mastication of chewing gum with regard to gender difference. J Prosthodont Res. 2009;53(1):11‐14. 10.1016/j.jpor.2008.08.002 19318065

[19] Watanabe A , Shiga H , Kobayashi Y . Occlusal contacting condition and masticatory function of 2 types of pattern that differ in the closing path of the mandibular incisal point during chewing. J Prosthodont Res. 2011;55(4):243‐247. 10.1016/j.jpor.2011.03.004 21531190

[20] Kuramochi A , Shiga H . Effect of denture treatment on masticatory movement in patients with complete dentures. J Prosthodont Res. 2019;63(2):245‐249. 10.1016/j.jpor.2018.12.005 30692048

[21] Lin C‐S , Wu C‐Y , Wu S‐Y , et al. Age‐related difference in functional brain connectivity of mastication. Front Aging Neurosci. 2017;9:82. 10.3389/fnagi.2017.00082 28420981 PMC5376560

[22] Lin CS , Wu SY , Wu CY , Ko HW . Gray matter volume and resting‐state functional connectivity of the motor cortex‐cerebellum network reflect the individual variation in masticatory performance in healthy elderly people. Front Aging Neurosci. 2015;7:247. 10.3389/fnagi.2015.00247 26779015 PMC4703716

[23] Miller SC , Textbook of Peridontia, 2 ed. Philadelphia: The Blaskiston Co.; 1947:102‐103.

[24] Shiga H , Ishikawa A , Nakajima K , Tanaka A . Relationship between masticatory performance using a gummy jelly and food intake ability in Japanese complete denture wearers. Odontology. 2015;103(3):356‐359. 25183492 10.1007/s10266-014-0170-5

[25] Sakayori T , Maki Y , Hirata S , Okada M , Ishii T . Evaluation of a Japanese “Prevention of Long‐term Care” project for the improvement in oral function in the high‐risk elderly. Geriatrics & Gerontology International. 2013;13(2):451‐457. 10.1111/j.1447-0594.2012.00930.x 22963330

[26] Butler SG , Stuart A , Leng X , et al. The relationship of aspiration status with tongue and handgrip strength in healthy older adults. J Gerontol A Biol Sci Med Sci. 2011;66(4):452‐458. 21300744 10.1093/gerona/glq234 PMC3107020

[27] Hayashi R , Tsuga K , Hosokawa R , Yoshida M , Sato Y , Akagawa Y . A novel handy probe for tongue pressure measurement. Int J Prosthodont. 2002;15(4):385‐388. 12170854

[28] Kimura M , Mizuta C , Yamada Y , Okayama Y , Nakamura E . Constructing an index of physical fitness age for Japanese elderly based on 7‐year longitudinal data: sex differences in estimated physical fitness age. Age. 2012;34(1):203‐214. 21424789 10.1007/s11357-011-9225-5 PMC3260370

[29] Faul F , Erdfelder E , Lang AG , Buchner A . G*Power 3: a flexible statistical power analysis program for the social, behavioral, and biomedical sciences. Behav Res Methods. 2007;39(2):175‐191. 17695343 10.3758/bf03193146

